# Artificial Intelligence Adoption in Orthopedics: A Multicenter International Survey From Germany and Greece

**DOI:** 10.7759/cureus.101374

**Published:** 2026-01-12

**Authors:** Sosanna Ierodiaconou, Amadeo Touet, Henry Pennig, Kristian Welle, Sebastian Scheidt, Christof Burger, Alexander Seuser

**Affiliations:** 1 Department of Orthopedics and Trauma Surgery, Karamandaneio Prefecture Children Hospital of Patras, Patras, GRC; 2 Department of Orthopedics and Trauma Surgery, University Hospital Bonn, Bonn, DEU

**Keywords:** ai in diagnostics, ai integration, artificial intelligence (ai), clinical decision support, data privacy, healthcare systems, multicenter survey study, personalised treatment

## Abstract

Integrating artificial intelligence (AI) into healthcare systems is rapidly transforming medical practice, particularly in diagnostics, personalized treatment, and clinical workflow optimization. This multicenter international survey investigates the adoption, current usage, and perceived benefits of AI technologies within healthcare systems. A total of 28 participants responded, including 13 from German university hospitals, six from Greek university hospitals, and nine general healthcare providers in Greece. Respondents from German university hospitals reported more frequent use of AI, whereas insufficient training and concerns about data security were more prominent barriers in Greece. Previous studies highlight the growing role of AI in enhancing diagnostic accuracy through machine learning algorithms, supporting decision-making in treatment plans, and automating administrative tasks to alleviate clinician workload. Despite its promising potential, the survey identifies several barriers, including concerns about data privacy and security, the integration of AI into existing infrastructure, and the need for regulatory frameworks.

## Introduction

The term artificial intelligence (AI) was first introduced by John McCarthy and refers to the application of algorithms that enable machines to solve problems that traditionally required human intelligence [1-3]. AI in healthcare refers to integrating advanced technologies such as machine learning, natural language processing, and data analytics to improve the efficiency and effectiveness of the healthcare system. Vast amounts of medical data are analyzed, so that AI can help healthcare providers make informed decisions, improve patient care, and optimize resources, by suggesting personalized treatments, detecting diseases early, streamlining workflows, and improving outcomes, ultimately contributing to better healthcare services and experiences for patients [4,5]. The impact of AI is particularly evident in areas such as medical imaging, where machine learning algorithms can identify patterns in images that are often imperceptible to the human eye [6-8]. It can also be used in the emergency room as an aid to physicians in the detection of fractures that may have been otherwise missed [5,8,9]. It has even been introduced into the field of psychology, proposing advanced virtual human avatars that can conduct the conversations needed to diagnose and treat patients with mental illness [10].

In addition, the integration of AI into administrative processes, such as appointment scheduling and billing, is reducing clinician burnout by automating routine tasks [1]. Moreover, AI can contribute to detecting and predicting pandemics, drug discovery, matching suitable patients to clinical trials, and many more [4]. Despite these advances, several barriers remain, including data privacy concerns, the integration of AI systems into existing healthcare infrastructure, and regulatory and ethical considerations [11,12]. Although the literature on AI in healthcare is expanding, limited evidence exists regarding how orthopedic surgeons perceive AI adoption across different healthcare settings. Furthermore, no comparative data are available between Germany and Greece concerning clinicians’ familiarity with and attitudes toward AI. This study examines the adoption and integration of AI in healthcare through a multicenter international survey conducted across three hospitals in Germany and Greece, with the main objectives to assess AI familiarity, current clinical applications, perceived benefits, ethical and technical concerns, and compare perspectives among orthopedic and trauma clinicians across institutions.

## Materials and methods

This multicenter, cross-sectional survey was conducted in accordance with the consensus-based checklist for reporting of survey studies (CROSS) to ensure methodological transparency and reproducibility. The study aimed to assess the familiarity, usage, and perception of AI among orthopedic and trauma surgeons and residents. The study was conducted across three hospitals - one German university hospital and two Greek hospitals, comprising one university hospital and one general hospital - between January 1 and February 11, 2025. The questionnaire was available for approximately one month, as this timeframe was sufficient to achieve the predefined target sample size among orthopedic trauma surgeons and residents. Response rates plateaued toward the end of the collection period, indicating response saturation. Additionally, limiting the survey duration helped minimize temporal bias related to variations in clinical workload, rotation schedules, and trauma volume, thereby ensuring consistency in participants’ clinical context at the time of response.

The questionnaire was developed using Google Forms and was made available in three equivalent language versions: one in German for participants at the German university hospital and two identical versions translated into English for participants at the Greek hospitals (Appendix A for the German version of the full instrument and Appendices B and C for the English version of the full instrument). It consisted of 10 questions covering six thematic areas: familiarity and current use of AI, applications of AI in diagnostics and therapy planning, ethical and legal considerations, technical challenges, perceived efficiency and optimization of hospital processes, and future perspectives. All questions were mandatory, with most presented as multiple-choice or Likert-scale items, and optional free-text fields were included to allow respondents to provide qualitative comments.

Participation was voluntary and anonymous. The questionnaire was distributed to all members - surgeons and residents - of the orthopedic and trauma departments at the participating hospitals: 55 clinicians from the German university hospital, 22 from the Greek university hospital, and 10 from a general hospital in Greece, a total of 87 clinicians. A total of 28 (32.2%) orthopedic clinicians completed the survey, including 13 of 55 (23.6%) from the German university hospital, six of 22 (27.3%) from the Greek university hospital, and nine of 10 (90%) from the general hospital in Greece. In the Greek cohort, the term "general healthcare providers" refers to orthopedic trauma surgeons practicing in general (non-tertiary) hospitals, rather than non-orthopedic healthcare professionals. Electronic informed consent was obtained before survey completion, and no incentives were provided. Data were collected electronically and automatically recorded in Google Sheets, with the “one response per participant” feature enabled to prevent duplicate entries.

Descriptive statistics were used to summarize participant characteristics and response frequencies, and comparative analyses were performed to identify differences between the participating hospitals. Although free-text responses were permitted for selected questions, no qualitative responses were received.

As no patient data or clinical interventions were involved, the study was exempt from formal ethical approval under institutional regulations. All participation was voluntary and anonymous, and data handling complied with the General Data Protection Regulation (GDPR).

## Results

The data show differences in familiarity with AI across the participating groups. A total of 28 orthopedic surgeons participated in the survey and responded to the questionnaire, including 13 out of 55 (23.6%) from German university hospitals, six out of 22 (27.3%) from Greek university hospitals, and nine out of 10 (90%) general healthcare providers in Greece. The findings are primarily descriptive due to small sample sizes. In Germany's university hospitals, 61.5% (8/13) of respondents reported being "somewhat familiar" with AI, compared to 50% (3/6) in the Greek university hospitals and a mere 11.1% (1/9) among general healthcare providers in Greece. A higher percentage of respondents in Germany's university hospitals (7.7%, 1/13) indicated being "very familiar" with AI, compared to 0% in both Greek university hospitals and general healthcare providers in Greece.

**Figure 1:**
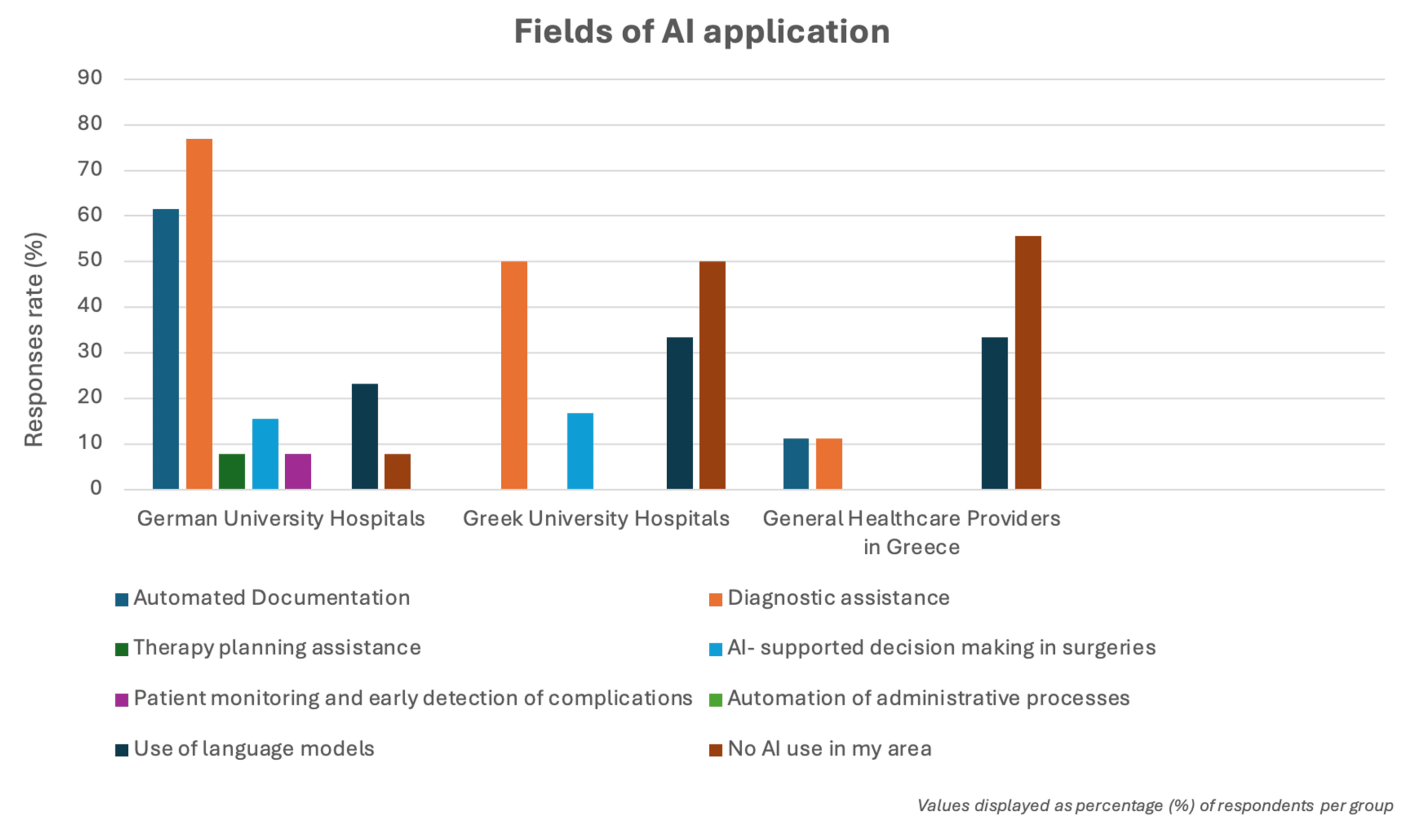
Fields of artificial intelligence (AI) application German university hospitals lead in AI adoption - mainly for diagnostics (76.9%, 10/13) and documentation (61.5%, 8/13) - while Greek university hospitals apply AI primarily in diagnostics (50%, 3/6), and general healthcare providers in Greece report limited AI use, with many having none at all (55.6%, 5/9).

In terms of AI use in clinical practice, German university hospitals reported higher adoption rates, with 76.9% (10/13) of participants reporting the use of AI for medical imaging, compared with 50% (3/6) in Greek university hospitals and only 11.1% (1/9) among general healthcare providers in Greece. Additionally, 61.5% (8/13) of respondents in German university hospitals reported using AI for automated documentation, while respondents in Greek university hospitals reported no use of AI for this purpose (Figure 1).

**Figure 2:**
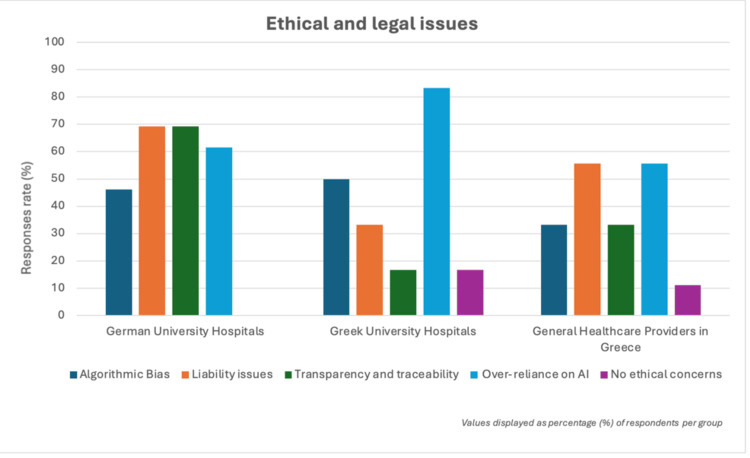
Ethical and legal issues German university hospitals are most concerned with liability (69.2%, 9/13) and transparency (69.2%, 9/13), Greek university hospitals highlight over-reliance on AI as the main issue (83.3%, 5/6), while general healthcare providers in Greece emphasize liability (55.6%, 5/9) and over-reliance (55.6%, 5/9), with one participant reporting no ethical concerns (11.1%, 1/9).

Despite the differences in the overall adoption rates, similarities in ethical concerns were observed across groups. In both German and Greek university hospitals, respondents expressed concerns about algorithmic bias. Specifically, 46.2% (6/13) of respondents in Germany's university hospitals and 50% (3/6) in Greek university hospitals expressed concerns about the risk of algorithmic bias in AI systems. Additionally, 69.2% (9/13) of respondents from German university hospitals and 16.7% (1/6) from Greek university hospitals emphasized the need for transparency and accountability in AI decision-making processes (Figure 2).

**Figure 3:**
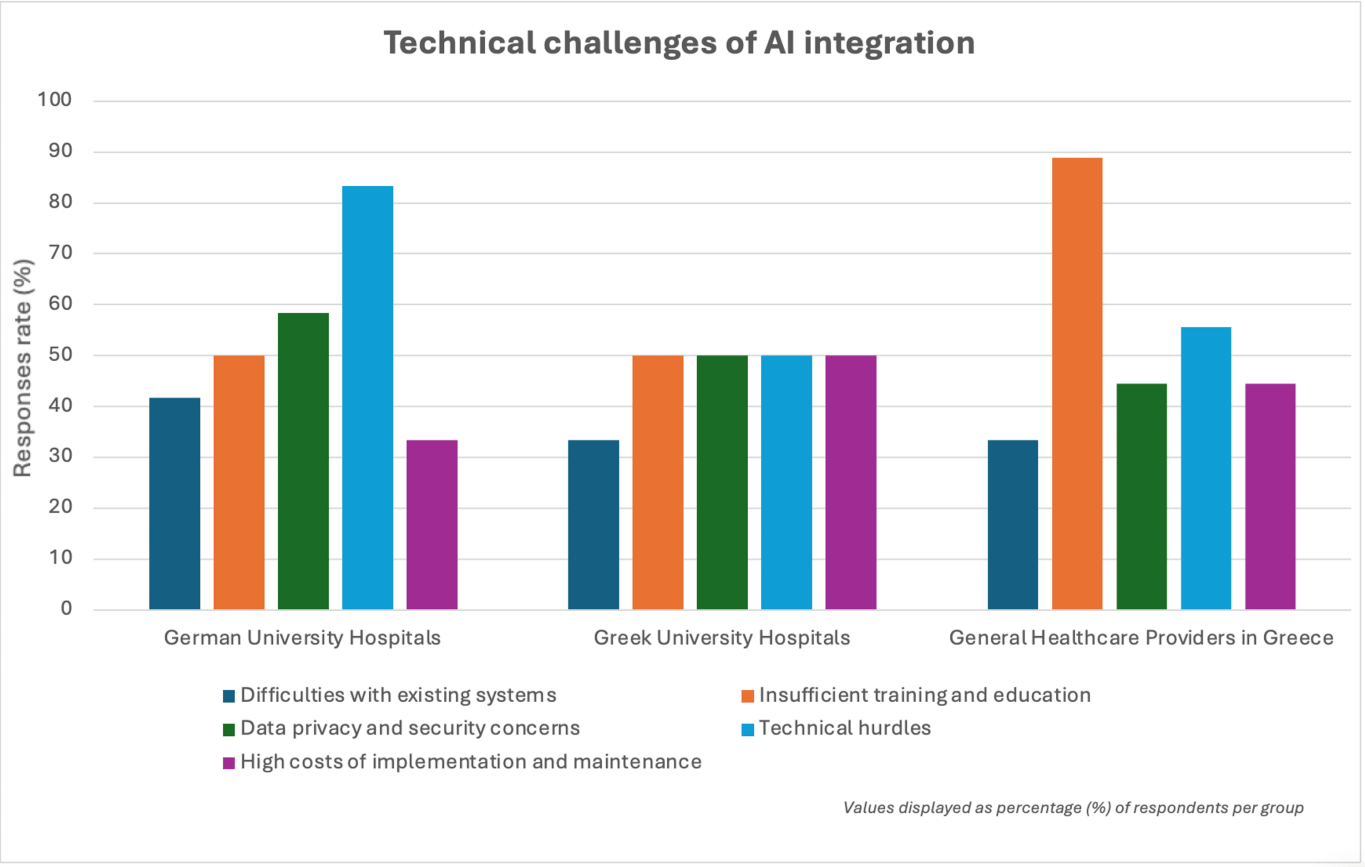
Technical challenges of AI integration German university hospitals see technical hurdles (83.3%, 10/13) and data security (58.3%, 7/13) as the main barriers to AI. Greek university hospitals face evenly distributed challenges, including training (50%, 3/6), technical hurdles (50%, 3/6), high costs (50%, 3/6), and security (50%, 3/6), while general healthcare providers in Greece struggle primarily with insufficient training and education (88.9%, 8/9).

Furthermore, concerns about data privacy were reported in both German university hospitals and general healthcare providers in Greece, with 44.4% (4/9) of the latter expressing concerns about data security, mirroring the findings from Germany’s university hospitals (58.3% of respondents, 7/13) (Figure 3). The survey also showed that training and education gaps were viewed as one of the main barriers to the effective use of AI in Greece, particularly in general healthcare providers, where 88.9% (8/9) of respondents indicated that insufficient training was a significant issue (Figure 3).

**Figure 4:**
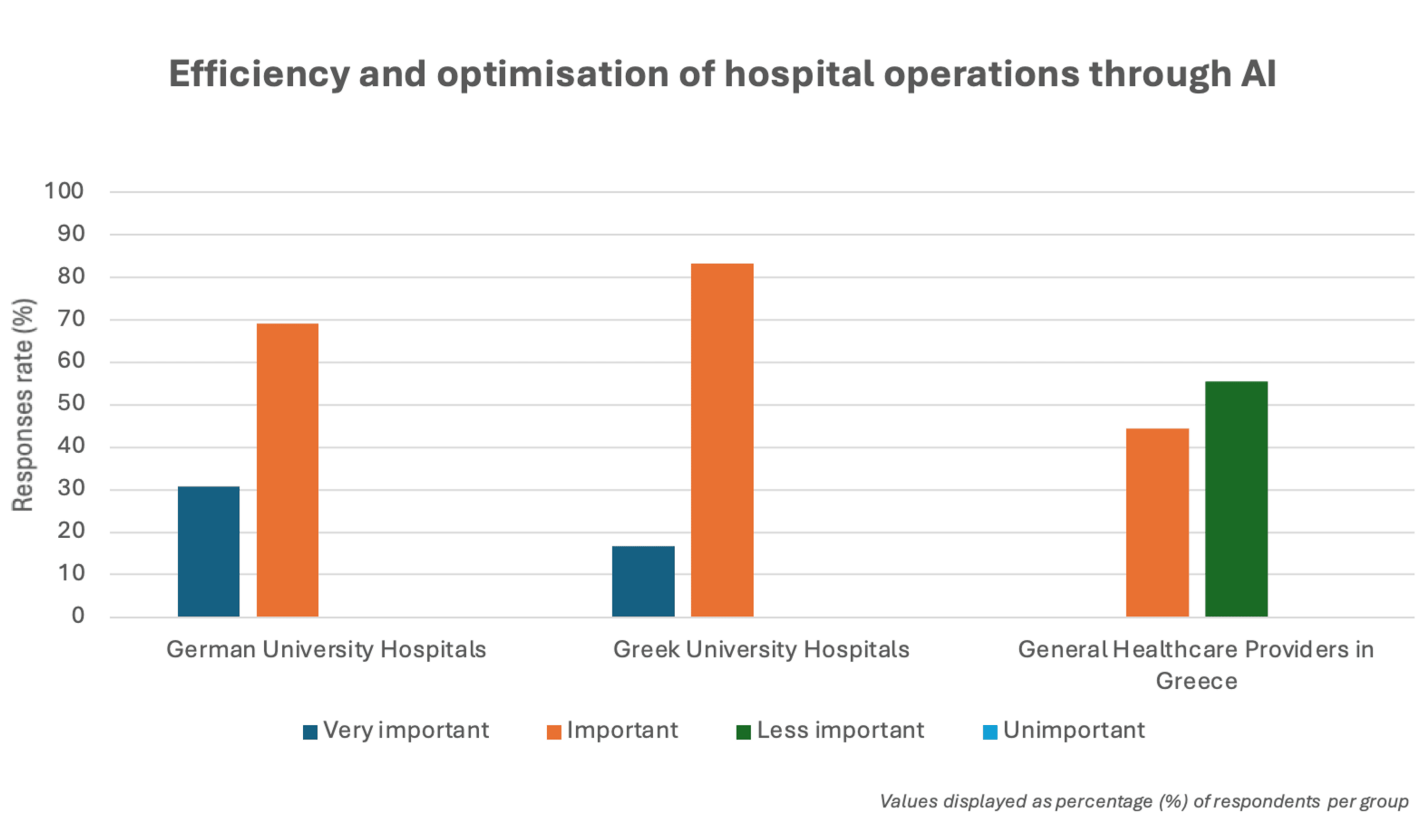
Efficiency and optimization of hospital operations through AI Perceptions of AI’s role in optimizing hospital operations across healthcare institutions. Both German and Greek university hospitals predominantly view AI as "important" (69.2%, 9/13, and 83.3%, 5/6, respectively) or "very important" (30.8%, 4/13, and 16.7%, 1/6, respectively), with the latter showing slightly higher emphasis on importance. In contrast, general healthcare providers in Greece display a more moderate view, considering AI to be largely "important" (44.4%, 4/9) or "less important" (55.6%, 5/9).

There is a growing recognition of AI’s potential to improve hospital operations across both countries. In Greece, 44.4% (4/9) of healthcare providers view AI as "important" for improving hospital operations and resource management. In Germany, 69.2% (9/13) of respondents from university hospitals share this view, indicating a higher level of recognition. Greek university hospitals show even stronger support, with 83.3% (5/6) considering AI "important" for optimizing operations. These findings underscore the growing awareness of AI's role in improving healthcare efficiency and patient care (Figure 4).

## Discussion

The survey demonstrated differences in the adoption and use of AI between Germany and Greece, particularly when comparing university hospitals with general hospital settings. However, these findings should be interpreted as exploratory and primarily descriptive. AI adoption appeared more advanced in the German cohort, within university hospitals, where established technological infrastructure facilitates integration into clinical workflows. In contrast, orthopedic trauma surgeons practicing in general hospitals in Greece reported greater challenges related to AI adoption and familiarization. These trends suggest possible disparities in AI readiness, but direct country-level comparisons should be treated as preliminary, particularly since general healthcare providers were included only in Greece, limiting comparability.

Ethical concerns are widespread in both countries. Respondents from university hospitals in Germany raised concerns about liability issues related to decisions made by AI systems (Figure 2). These concerns reflect broader issues, such as algorithmic bias, where AI systems may unintentionally disadvantage certain patient groups and nationalities [3,4,12,13]. For example, the prevalence of some diseases differs between Western and Eastern countries, so there is less data on disease profiles that are more common in Eastern countries but less so in Western countries. This is also the case with the approach to treating some diseases [13]. Additionally, general healthcare providers in Greece reported particularly great concerns regarding data security and patient confidentiality, emphasizing the importance of regulatory oversight in the integration of AI with sensitive patient data. Access to sensitive medical information raises concerns about patient privacy [3]. The need for strong regulatory frameworks to prevent misuse or discriminatory practices is acknowledged across both Germany and Greece. This is in agreement with the findings of the study conducted by Sunarti et al. that considered the opportunities and risks of integrating AI into the healthcare system, concluding that although the role of AI in healthcare management, medical decision-making, diagnosis, and patient treatment is promising, numerous challenges regarding safety, efficacy, privacy, and data bias need to be addressed. Therefore, these findings once again highlight the challenges of integrating AI into the healthcare system on a global scale [4,14,15].

Regarding barriers to adoption, Germany faces fewer challenges due to better infrastructure and more resources for AI integration [11]. The higher level of AI adoption in Germany's university hospitals is facilitated by greater access to AI tools, in fields such as medical imaging, automated documentation, and decision support systems. In Greece, healthcare providers report challenges, highlighting that insufficient training in integrating and using AI is a key barrier to progress (Figure 3). Due to varying levels of technology literacy and limited hands-on experience using AI applications in practice, some healthcare professionals are not sufficiently comfortable with digital technology, as they can face challenges in learning to integrate and use technology. Hence, this may further limit the integration of AI in healthcare [16]. Furthermore, Sun and Medaglia and Wubineh et al. pointed out that there is often a lack of public awareness and understanding of the potential benefits and limitations of AI in healthcare, leading to unrealistic expectations that can make these technologies difficult to adopt by physicians [13,16].

Civaner et al. surveyed the perceptions of medical students in Turkey regarding the potential impact of AI on medicine and their thoughts on the AI topics that should be included in the medical curriculum. They concluded that students still do not receive structured or standardized education about AI, despite their eagerness to learn more about its future benefits and potential risks [17]. Therefore, more training programs and education initiatives should be added to the medical school curriculum to bridge the gap in AI literacy [17,18].

Lee and Yoon bring to the forefront the issue of possible job losses, as the integration of AI could lead to the replacement of some healthcare providers due to automation, with radiologists potentially being the first to be replaced. However, this view does not consider that the replacement of medical professionals is unlikely to happen any time soon. Rather, AI is expected to serve as a support tool that relieves the burden on healthcare providers, particularly by assisting in tasks like medical imaging interpretation. Such AI-based support tools will still require physician supervision to ensure accurate diagnoses and patient care. Furthermore, the integration of AI will likely create many new roles to manage and implement these systems and devices [18].

The importance of AI in optimizing hospital operations and resources, such as appointment scheduling, bed occupancy, management of operating rooms, and staff planning, was widely acknowledged across the survey responses. The findings are in line with the broader literature, which emphasizes the critical role of AI in improving hospital efficiency and resource allocation [7,19]. Studies, such as those by Brynjolfsson and McAfee, show how AI can be effectively used to automate routine administrative tasks, helping hospitals better manage appointments, optimize bed usage, and plan staff schedules [1]. Bellini et al. conducted a study on the role of AI, specifically machine learning, in operating room management, emphasizing its contribution to surgical case duration prediction, resource allocation, and surgical case cancellations detection [20,21]. The results from the surveys further support these claims, underlining AI's importance in reducing administrative burdens and improving hospital resource management [1,20,21].

Several methodological limitations constrain the generalizability of this study. Its main limitation is the relatively small sample size within each group, comprising 13 respondents from a German university hospital, six from a Greek university hospital, and nine orthopedic trauma surgeons practicing in a general hospital in Greece. To obtain a more comprehensive and representative understanding of AI applications and integration in healthcare, future studies should include larger and more diverse participant populations. Additionally, as with all voluntary, anonymous survey-based studies, some degree of selection and non-response bias is possible. Finally, because the survey was conducted in only three hospitals, two of which were located in the same country, the findings may reflect institution- or region-specific practices and may not be fully generalizable to orthopedic trauma providers in other healthcare settings. Despite these constraints, the study’s strengths include its multicenter international design, inclusion of both university and general hospital settings, and focus on orthopedics and traumatology - a field where AI adoption remains understudied.

## Conclusions

The adoption of AI in healthcare shows promise in supporting improvements in diagnostic accuracy, treatment personalization, and clinical workflow optimization. However, in this exploratory survey, observed trends suggest differences in AI adoption and use across the participating settings. Respondents from Germany reported more advanced AI adoption and integration, particularly in university hospitals, whereas respondents from Greece, especially those practicing in general hospital settings, reported greater challenges related to infrastructure, data security considerations, and resource availability. Given the exploratory nature of this study and the small sample sizes, these findings should be interpreted with caution. To help address existing challenges and support the responsible use of AI, continued investment in AI education, development of clear regulatory frameworks, and increased international collaboration may be beneficial. Principles such as fairness, transparency, trustworthiness, accountability, privacy, and empathy remain essential for the responsible implementation of AI. Maintaining an appropriate balance between human expertise and AI-supported systems is important to ensure ethical and effective integration that benefits both patients and healthcare providers.

## Appendices

Appendix A

Fragebogen zur Nutzung von Künstlicher Intelligenz im Klinikalltag: Einblicke aus der Orthopädie und Unfallchirurgie

Liebe Befragte,

Künstliche Intelligenz (AI) gewinnt zunehmend an Bedeutung, auch im Gesundheitswesen. Sie bietet großes Potenzial, um Diagnosen zu verbessern, Therapien zu personalisieren und Abläufe zu optimieren.

Mit dieser Umfrage möchten wir erfahren, wie AI aktuell in Ihrem Klinikalltag genutzt wird und welches Potenzial Sie in der Technologie sehen. Ihre Teilnahme hilft uns, die Chancen und Herausforderungen von AI im Gesundheitswesen besser zu verstehen.

Vielen Dank für Ihre Unterstützung!

Abschnitt 1: Allgemeines Verständnis und Nutzung von AI in der Klinik

1. Wie vertraut sind Sie mit dem Thema Künstliche Intelligenz (AI) in der medizinischen Praxis?

Sehr vertraut - Ich habe umfassende Kenntnisse und setze AI aktiv ein

Etwas vertraut - Ich habe grundlegende Kenntnisse, aber keine tiefgehende Erfahrung

Kaum vertraut - Ich habe nur wenig Wissen über AI in der Medizin

Gar nicht vertraut - Ich habe keine Kenntnisse oder Erfahrung mit AI

2. In welchen Bereichen des Klinikalltags haben Sie persönlich den Einsatz von AI wahrgenommen oder genutzt? (Mehrfachantworten möglich)

Automatisierte Dokumentation (z.B. Spracherkennung für ärztliche Befunde)

Unterstützung in der Bilddiagnostik (z.B. Röntgen-, MRT-, CT-Bildanalyse)

Assistenz bei der Therapieplanung (z.B. personalisierte Behandlungsstrategien)

AI-gestützte Entscheidungshilfen in der Chirurgie (z.B. Operationsplanung, Navigationssysteme)

Patientenüberwachung und Früherkennung von Komplikationen (z.B. AI-gestützte Vitalzeichenanalyse)

Automatisierung von administrativen Prozessen (z.B. Terminplanung, Abrechnung)

Keinen Einsatz von AI in meinem Bereich festgestellt

Einsatz von Sprachmodellen zur Generierung von medizinischen Befunden und Unterstützung von Ärzten (z.B. ChatGPT, etc.)

Andere (bitte spezifizieren):_____________

3. Wie häufig verwenden Sie AI-unterstützte Systeme in Ihrem Arbeitsalltag?

Täglich - AI ist ein unverzichtbarer Teil meiner täglichen Arbeit

Mehrmals pro Woche - AI wird regelmäßig genutzt, aber nicht täglich

Selten - Ich setze AI nur gelegentlich ein

Nie - Ich nutze keine AI-Technologien in meiner Praxis

Abschnitt 2: AI in der Diagnostik und Therapie

4. Wie schätzen Sie die Bedeutung der AI bei der Optimierung der diagnostischen Prozesse in der Orthopädie und Unfallchirurgie ein?

Sehr hoch - AI hilft erheblich, Diagnosen zu beschleunigen und zu präzisieren (z.B. durch Bildanalyse)

Mäßig hoch - AI trägt zur Diagnose bei, aber es gibt noch viele manuelle Eingriffe

Gering - AI hat derzeit einen sehr begrenzten Einfluss auf diagnostische Prozesse

Kein Einfluss - Ich nutze keine AI zur Diagnostik

5. Wie bewerten Sie die Rolle von AI in der Personalisierung von Behandlungsplänen in der Orthopädie und Unfallchirurgie?

Sehr positiv - AI ermöglicht eine maßgeschneiderte Therapie, die die besten Ergebnisse liefert

Positiv - AI bietet wertvolle Unterstützung bei der Auswahl individueller Therapien

Neutral - AI hat noch keinen signifikanten Einfluss auf die Personalisierung der Behandlung

Negativ - AI kann nicht alle individuellen Bedürfnisse eines Patienten berücksichtigen

Keine Meinung - Ich nutze AI noch nicht in der Therapieplanung

Abschnitt 3: Ethische und rechtliche Fragestellungen

6. Wie sehen Sie die ethischen Herausforderungen bei der Nutzung von AI in der medizinischen Diagnose und Therapie?

Algorithmische Voreingenommenheit - AI-Systeme könnten bestimmte Patientengruppen benachteiligen

Haftungsfragen - Wer ist verantwortlich, wenn ein AI-gestütztes System eine falsche Diagnose oder Therapie empfiehlt?

Transparenz und Nachvollziehbarkeit - Die Entscheidungsprozesse der AI sind für Ärzte und Patienten nicht immer nachvollziehbar

Übermäßige Abhängigkeit von AI - Ärzte könnten in ihrer Urteilsfähigkeit beeinträchtigt werden, wenn sie sich zu sehr auf AI verlassen

Keine ethischen Bedenken - Ich sehe keine ethischen Probleme bei der Nutzung von AI

Andere (bitte spezifizieren):_______________

Abschnitt 4: Technische Herausforderungen und Integration von AI

7. Welche speziellen Herausforderungen sehen Sie bei der Implementierung von AI-Technologien in Ihre klinischen Arbeitsabläufe?

Schwierigkeit der Integration in bestehende Systeme (z.B. elektronische Patientenakten)

Unzureichende Schulung und Weiterbildung für das medizinische Personal

Datenschutz- und Sicherheitsbedenken im Umgang mit sensiblen Patientendaten

Technische Hürden und Inkompatibilität zwischen AI-Tools und vorhandener Kliniksoftware

Hohe Kosten der Implementierung und Wartung von AI-Systemen

Andere (bitte spezifizieren):_______________

Abschnitt 5: Effizienz und Optimierung von Krankenhausabläufen durch AI

8. Wie wichtig ist die Unterstützung durch AI für die Optimierung von Krankenhausabläufen und -ressourcen (z.B. Terminplanung, Bettenbelegung, Personalplanung)?

Sehr wichtig - AI trägt wesentlich zur Verbesserung der Effizienz und Ressourcenplanung bei

Wichtig - AI unterstützt die Optimierung in einigen Bereichen, könnte jedoch noch mehr leisten

Weniger wichtig - Es gibt andere, effizientere Lösungen zur Optimierung von Krankenhausabläufen

Unwichtig - AI spielt keine Rolle in der Optimierung von Abläufen und Ressourcen

Abschnitt 6: Zukunftsperspektiven und Empfehlungen

9. Welche weiteren Empfehlungen würden Sie zur Förderung des verantwortungsvollen Einsatzes von AI im Gesundheitswesen geben?

Strengere gesetzliche Regelungen und Standards für die Nutzung von AI

Förderung von Transparenz in den Entscheidungsprozessen von AI-Systemen

Erweiterte Schulungs- und Fortbildungsprogramme für das medizinische Personal

Förderung von Forschung und Entwicklung im Bereich ethische AI

Keine weiteren Empfehlungen - Die aktuellen Maßnahmen reichen aus

10. Wie sehen Sie die Skalierbarkeit von AI-Lösungen in verschiedenen Klinikgrößen, insbesondere in kleinen und unterversorgten Einrichtungen?

Sehr hoch - AI-Lösungen können problemlos auf kleine und unterversorgte Einrichtungen angewendet werden

Mäßig hoch - Skalierbarkeit ist möglich, aber es gibt Herausforderungen, insbesondere im Hinblick auf Infrastruktur und Kosten

Niedrig - AI-Lösungen sind in kleinen oder unterversorgten Einrichtungen schwer umzusetzen

Keine Meinung - Ich habe keine Erfahrung mit der Skalierbarkeit von AI in kleineren Einrichtungen

Appendix B

Survey on the Use of Artificial Intelligence in Clinical Practice: Insights From Orthopedics and Trauma Surgery at a Greek University Hospital

Dear participant,

Artificial intelligence (AI) is becoming increasingly important, including in healthcare. In particular, it offers great potential for improving diagnostics, personalizing treatments, and optimizing workflows.

With this survey, we aim to understand how AI is currently being used in your clinical practice and what potential you see in this technology. Your participation will help us better understand the opportunities and challenges of AI in healthcare.

Thank you for your support!

Section 1: General Understanding and Use of AI in the Clinic

1. How familiar are you with the topic of Artificial Intelligence (AI) in medical practice? 

Very familiar - I have extensive knowledge and actively use AI.

Somewhat familiar - I have basic knowledge but no in-depth experience.

Slightly familiar - I have limited knowledge of AI in medicine.

Not familiar at all - I have no knowledge or experience with AI.

2. In which areas of the clinic have you personally observed or used AI? (Multiple answers possible) 

Automated documentation (e.g., voice recognition for medical reports, etc.)

Diagnostic assistance (e.g., X-ray, MRI, CT scan analysis, etc.)

Therapy planning assistance (e.g., personalized treatment strategies, etc.)

AI-supported decision-making in surgery (e.g., surgical planning, navigation systems, etc.)

Patient monitoring and early detection of complications (e.g., AI-supported vital signs analysis, etc.)

Automation of administrative processes (e.g., appointment scheduling, billing, etc.)

No AI use in my area

Use of language models for generating medical reports and assisting physicians (e.g., ChatGPT, etc.)

Other (please specify): _____________

3. How frequently do you use AI-supported systems in your daily work? 

Daily - AI is an indispensable part of my daily work.

Several times a week - AI is used regularly but not daily.

Rarely - I use AI only occasionally.

Never - I do not use AI technologies in my practice.

Section 2: AI in Diagnostics and Therapy

4. How do you assess the importance of AI in optimizing diagnostic processes in orthopedics and trauma surgery? 

Very high - AI significantly helps speed up and improve diagnoses (e.g., through image analysis, genetic tests).

Moderately high - AI contributes to diagnostics, but many manual interventions are still required.

Low - AI currently has a very limited impact on diagnostic processes.

No impact - I do not use AI for diagnostics.

5. How do you assess the role of AI in personalizing treatment plans in orthopedics and trauma surgery? 

Very positive - AI enables personalized treatment, leading to the best outcomes.

Positive - AI provides valuable assistance in selecting individual therapies.

Neutral - AI has not yet had a significant impact on personalized treatment.

Negative - AI cannot address all the individual needs of a patient.

No opinion - I do not use AI in therapy planning.

Section 3: Ethical and Legal Issues

6. What ethical challenges do you see in the use of AI in medical diagnosis and therapy?

Algorithmic bias - AI systems may disadvantage certain patient groups.

Liability issues - Who is responsible if an AI-supported system recommends a wrong diagnosis or therapy?

Transparency and traceability - The decision-making processes of AI are not always understandable for doctors and patients.

Over-reliance on AI - Doctors may be impaired in their judgment if they rely too much on AI.

No ethical concerns - I do not see any ethical problems with the use of AI.

Other (please specify): ____________

Section 4: Technical Challenges and Integration of AI

7. What specific challenges do you see in implementing AI technologies into your clinical workflows? 

Difficulties in integration with existing systems (e.g., electronic medical records)

Insufficient training and education for medical personnel

Data privacy and security concerns when handling sensitive patient data

Technical hurdles and incompatibility between AI tools and existing clinic software

High costs of implementing and maintaining AI systems

Other (please specify): ___________

Section 5: Efficiency and Optimization of Hospital Operations Through AI

8. How important is AI support for optimizing hospital operations and resources (e.g., appointment scheduling, bed occupancy, staff planning)?

Very important - AI greatly contributes to improving efficiency and resource planning.

Important - AI supports optimization in some areas but could do more.

Less important - There are other more efficient solutions for optimizing hospital operations.

Unimportant - AI plays no role in optimizing operations and resources.

Section 6: Future Perspectives and Recommendations

9. What additional recommendations would you suggest for the responsible use of AI in healthcare? 

Stricter regulations and standards for the use of AI

Promoting transparency in AI decision-making processes

Expanding training and education programs for medical personnel

Promoting research and development in the field of ethical AI

No further recommendations - Current measures are sufficient

10. How do you rate the scalability of AI solutions in different hospital sizes, especially in small and underserved facilities? 

Very high - AI solutions can be easily applied to small and underserved facilities.

Moderately high - Scalability is possible, but there are challenges, especially regarding infrastructure and costs.

Low - AI solutions are difficult to implement in small or underserved facilities.

No opinion - I have no experience with the scalability of AI in smaller facilities.

Appendix C

Survey on the Use of Artificial Intelligence in Clinical Practice: Insights from Orthopedics and Trauma Surgery at a General Hospital in Greece

Dear participant,

Artificial intelligence (AI) is becoming increasingly important, including in healthcare. In particular, it offers great potential for improving diagnostics, personalizing treatments, and optimizing workflows.

With this survey, we aim to understand how AI is currently being used in your clinical practice and what potential you see in this technology. Your participation will help us better understand the opportunities and challenges of AI in healthcare.

Thank you for your support!

Section 1: General Understanding and Use of AI in the Clinic

1. How familiar are you with the topic of artificial intelligence (AI) in medical practice? 

Very familiar - I have extensive knowledge and actively use AI.

Somewhat familiar - I have basic knowledge but no in-depth experience.

Slightly familiar - I have limited knowledge of AI in medicine.

Not familiar at all - I have no knowledge or experience with AI.

2. In which areas of the clinic have you personally observed or used AI? (Multiple answers possible)

Automated documentation (e.g., voice recognition for medical reports, etc.)

Diagnostic assistance (e.g., X-ray, MRI, CT scan analysis, etc.)

Therapy planning assistance (e.g., personalized treatment strategies, etc.)

AI-supported decision-making in surgery (e.g., surgical planning, navigation systems, etc.)

Patient monitoring and early detection of complications (e.g., AI-supported vital signs analysis, etc.)

Automation of administrative processes (e.g., appointment scheduling, billing, etc.)

No AI use in my area

Use of language models for generating medical reports and assisting physicians (e.g. ChatGPT, etc.)

Other (please specify): _____________

3. How frequently do you use AI-supported systems in your daily work?

Daily - AI is an indispensable part of my daily work.

Several times a week - AI is used regularly but not daily.

Rarely - I use AI only occasionally.

Never - I do not use AI technologies in my practice.

Section 2: AI in Diagnostics and Therapy

4. How do you assess the importance of AI in optimizing diagnostic processes in orthopedics and trauma surgery? 

Very high - AI significantly helps speed up and improve diagnoses (e.g., through image analysis, genetic tests).

Moderately high - AI contributes to diagnostics, but many manual interventions are still required.

Low - AI currently has a very limited impact on diagnostic processes.

No impact - I do not use AI for diagnostics.

5. How do you assess the role of AI in personalizing treatment plans in orthopedics and trauma surgery? 

Very positive - AI enables personalized treatment, leading to the best outcomes.

Positive - AI provides valuable assistance in selecting individual therapies.

Neutral - AI has not yet had a significant impact on personalized treatment.

Negative - AI cannot address all the individual needs of a patient.

No opinion - I do not use AI in therapy planning.

Section 3: Ethical and Legal Issues

6. What ethical challenges do you see in the use of AI in medical diagnosis and therapy?

Algorithmic bias - AI systems may disadvantage certain patient groups.

Liability issues - Who is responsible if an AI-supported system recommends a wrong diagnosis or therapy?.

Transparency and traceability - The decision-making processes of AI are not always understandable for doctors and patients.

Over-reliance on AI - Doctors may be impaired in their judgment if they rely too much on AI.

No ethical concerns - I do not see any ethical problems with the use of AI.

Other (please specify): ____________

Section 4: Technical Challenges and Integration of AI

7. What specific challenges do you see in implementing AI technologies into your clinical workflows? 

Difficulties in integration with existing systems (e.g., electronic medical records)

Insufficient training and education for medical personnel

Data privacy and security concerns when handling sensitive patient data

Technical hurdles and incompatibility between AI tools and existing clinic software

High costs of implementing and maintaining AI systems

Other (please specify): ___________

Section 5: Efficiency and optimization of hospital operations through AI

8. How important is AI support for optimizing hospital operations and resources (e.g., appointment scheduling, bed occupancy, staff planning)?

Very important - AI greatly contributes to improving efficiency and resource planning.

Important - AI supports optimization in some areas but could do more.

Less important - There are other more efficient solutions for optimizing hospital operations.

Unimportant - AI plays no role in optimizing operations and resources.

Section 6: Future Perspectives and Recommendations

9. What additional recommendations would you suggest for the responsible use of AI in healthcare?

Stricter regulations and standards for the use of AI

Promoting transparency in AI decision-making processes

Expanding training and education programs for medical personnel

Promoting research and development in the field of ethical AI

No further recommendations - Current measures are sufficient

10. How do you rate the scalability of AI solutions in different hospital sizes, especially in small and underserved facilities? 

Very high - AI solutions can be easily applied to small and underserved facilities.

Moderately high - Scalability is possible, but there are challenges, especially regarding infrastructure and costs.

Low - AI solutions are difficult to implement in small or underserved facilities.

No opinion - I have no experience with the scalability of AI in smaller facilities.
